# Achieving Simultaneous Enhancement of Strength and Ductility in Aluminum Matrix Composites Reinforced by Dual-Scale Hybrid Reinforcement via Friction Stir Processing

**DOI:** 10.3390/ma18204780

**Published:** 2025-10-19

**Authors:** Zikun Wang, Xianyong Zhu, Chen Wang, Xiong Xiao, Ke Zhang, Cheng Jiang, Jiaan Liu

**Affiliations:** 1 School of Mechanical and Aerospace Engineering, Jilin University, Changchun 130022, China; zkwang24@mails.jlu.edu.cn (Z.W.); chenwangowen@163.com (C.W.); xiaoxiong20@mails.jlu.edu.cn (X.X.); zhangke23@mails.jlu.edu.cn (K.Z.); jiangcheng@jlu.edu.cn (C.J.); 2 College of Materials Science and Engineering, Jilin University, Changchun 130022, China; liuja@jlu.edu.cn

**Keywords:** friction stir processing, aluminum matrix composites, dual-scale reinforcement, copper, titanium, microstructure

## Abstract

Overcoming the strength–ductility trade-off in conventional aluminum matrix composites (AMCs) remains a significant challenge. This study employs dual-scale hybrid reinforcement particles comprising micron-sized Cu and nano-sized Ti, alongside bimodal micro-sized pure Al powders as matrix fillers. The AMCs were fabricated through ball milling (BM) combined with multi-pass friction stir processing (FSP). The homogenously distributed hybrid reinforcement particles generate an integrated composite region consisting of both coarse-grained (CG) and fine-grained (FG) structures, demonstrating enhanced material characteristics. The interwoven network of coarse- and fine-crystalline domains constructs a heterogeneous architecture that enables simultaneous improvement in both strength and ductility properties. The micron-Cu acts as a skeletal support within the matrix, enhancing load transfer efficiency and effectively hindering dislocation motion. The nano-Ti and in situ intermetallics facilitate grain refinement via the pinning effect and promote heterogeneous nucleation, which contributes to stress dispersion and dislocation obstruction. The addition of dual-scale micron-sized pure Al powder particles promotes the formation of the heterogeneous architecture, which enhances the balancing of strength and ductility in the composite. Following compositing (Al_10_-5Cu-10Ti-10Al_20_), the alloy exhibits an ultimate tensile strength (UST) of 267 MPa, a hardness of 98 HV, and an elongation of 16.7%, representing increases of 193.4%, 226.7%, and 9.9%, respectively, relative to the base metal.

## 1. Introduction

Aluminum alloys have the advantages of low density and strong machinability, which show the potential for lightweight applications. AMCs as a major research direction, can significantly enhance the strength of the matrix. Traditional reinforcements are usually ceramic particles, which have poor wettability with the matrix [1]. The strength enhanced by ceramic particles is usually accompanied by a decrease in ductility. Unlike ceramic particles, metal particles such as Ti, Fe, and Cu offer better comprehensive mechanical properties and wettability. Moreover, they can form intermetallic compounds with the aluminum matrix like Al_3_Ti [2], Al_3_Fe [3], and Al_2_Cu [4] at relatively lower temperatures. The intermetallic compounds enhance the interfacial bonding strength, which facilitates more efficient load transfer. Among all metal particles, Cu can undergo interfacial reactions with the matrix at relatively low temperatures, which helps to refine the grain structure of AMCs. The in situ formation of the brittle Al_2_Cu significantly enhances the strength but is not conducive to improving ductility [5].

Traditional composite fabrication techniques include casting [6], powder metallurgy [7], and selective laser melting [8], which are typically performed above the melting point of Al. These conventional methods face challenges such as the dispersion of reinforcement and the formation of harmful phases. Thus, the researchers focus more on solid-state rapid prototyping methods for fabricating composites below the melting point.

FSP originated from friction stir welding, which is a solid-state welding technique [9]. A non-consumable rotation tool is inserted into the material for processing. The tool consists of a shoulder and a pin. The heat generated by friction between the shoulder and the material is conducive to softening the matrix to facilitate plastic flow. Severe plastic deformation (SPD) promotes the plastic flow of the material to disperse reinforcement uniformly. SPD combined with high temperature in the stir zone (SZ) triggers dynamic recrystallization (DRX) to refine grain structures [10]. Therefore, the solid-state processing characteristics avoid the segregation and porosity defects associated with the casting method [11,12]. The low-temperature process prevents harmful interfacial reactions. Additionally, it features environmental friendliness, energy saving, low cost, and near-net-shape forming [13]. In recent years, many studies have attempted to fabricate metal particle-reinforced AMCs by FSP. Yadav et al. identified that intermetallic compounds such as Al_2_Cu can be formed during FSP [14]. Huang et al. investigated increasing the passes of FSP promoted the in situ reaction between the matrix and Cu [15]. The in situ Al_2_Cu significantly enhances the strength of AMCs. However, its inherent brittleness can induce stress concentration sites, which show limited effectiveness in enhancing ductility. Compared to other reinforcements, Ti shows a plastic deformation capacity and good compatibility with Al, which helps to improve the fracture toughness of the AMCs [16]. Additionally, the thermal expansion coefficient of Ti is relatively close to that of Al, which helps to avoid a stress concentration at the interface [17]. Huang et al. reported that the Ti distributed in the matrix and the formed Al_3_Ti intermetallic compounds promote grain refinement and strength enhancement [18]. Shen et al. found that Ti could form a strong interfacial bond with the matrix, which effectively bore loads and prevented stress concentration and crack initiation [16]. Adetunla et al. proposed that increasing the number of processing passes was beneficial to disperse TC4 particles [19].

Compared with microparticles, nanoparticles can effectively impede dislocation movement and refine the grain structure, while also improving stress distribution within the matrix [20]. The effect of nanoparticles with a high specific surface area in the matrix is mainly affected by their dispersibility. Early studies often directly used FSP to disperse nanoparticles. However, the dispersion effect was limited due to the short duration of the process. BM is a common pretreatment method that can disperse the reinforcement and refine the grain size effectively. The collisions between balls and powders during rotation promote interface bonding between reinforcement and the matrix. Comparing the milled and unmilled powder, Shyam found that BM significantly dispersed metal particles uniformly and embedded them on the surface of Al powder [21]. Morteza et al. indicated that BM before FSP facilitates the formation of intermetallic compounds [22]. Moreover, the numerous defects generated during BM increase the internal energy of the material, promoting the diffusion and reaction of metal particles during FSP [23].

Hybrid reinforcement particles are composed of two or more types of reinforcement, which leverage the individual strengths of each type within the matrix to achieve comprehensive enhancements in the properties of the composite material. Ramesh et al. utilized B_4_C combined with ZrB_2_ forming multi-scale reinforcements. The synergistic effect of the high hardness of B_4_C and the lubricity of ZrB_2_ significantly enhances the wear resistance of AMCs [24]. Zhang et al. found that multipass FSP improved the distribution of hybrid reinforcements, thereby improving the microstructure and mechanical properties [25]. Compared with single-type reinforcement, hybrid reinforcement systems composed of micro-nano particles significantly improve composite performance through multi-scale synergistic mechanisms. Xu et al. reported that micro-nano dual-scale hybrid particles further promote grain refinement and reduce stress concentration [26]. Guan et al. observed that the alternating distribution of coarse and fine grains formed by micron- and nano-sized SiC in the matrix creates an interlaced “soft–hard” structure, which promotes the uniformity of deformation [27]. In summary, the micro-nano dual-scale hybrid reinforcements play their respective roles in the microstructure and mechanical properties. Their synergistic effect achieves a balance between the strength and ductility of AMCs, which overcome the inherent trade-off between these properties in traditional AMCs with a single grain size.

Few studies focus on the effects of AMCs reinforced by multi-scale hybrid metal particles. In this study, the AMCs reinforced by micro Cu combined with nano Ti were fabricated through BM and subsequent multipass FSP. Al 1060 was selected as the base metal to demonstrate the in situ intermetallic and strengthening mechanisms while minimizing the impact of other elements [28]. The synergistic effects of dual-scale hybrid metal particles on the microstructure and mechanical properties were derived by comparison with single-scale particles.

Addressing the urgent demand for next-generation lightweight structures that simultaneously require high strength and high ductility, the present work proposes a “dual-scale Cu/Ti hybrid-reinforcement + multi-pass FSP” strategy that is readily transferable to engineering components such as aircraft fuselage panels, automotive chassis brackets, and high-speed train bogie weld joints. Future efforts should focus on tailoring the spatial distribution of in situ intermetallics and integrating additive manufacturing for near-net shaping of complex geometries, thereby accelerating the adoption of advanced AMCs in high-end equipment.

## 2. Materials and Methods

The substrate and cover plate were both constructed from commercial Al 1060, with dimensions of 200 mm × 75 mm × 8 mm and 2 mm, respectively. Table 1 shows the elemental composition of the substrate. Commercially pure Al powders (99.7% purity, sourced from Changsha Tianjiu Co. Ltd., Changsha, China), micro Cu and nano Ti (99.5% purity, supplied by Shanghai Maoguo Nano Co. Ltd., Shanghai, China) were measured with an average particle size of 7.8 µm (fine-particle Al), 17.3 µm (coarse-particle Al), 2.1 µm (Cu), and 68.8 nm (Ti).

**Figure 1:**
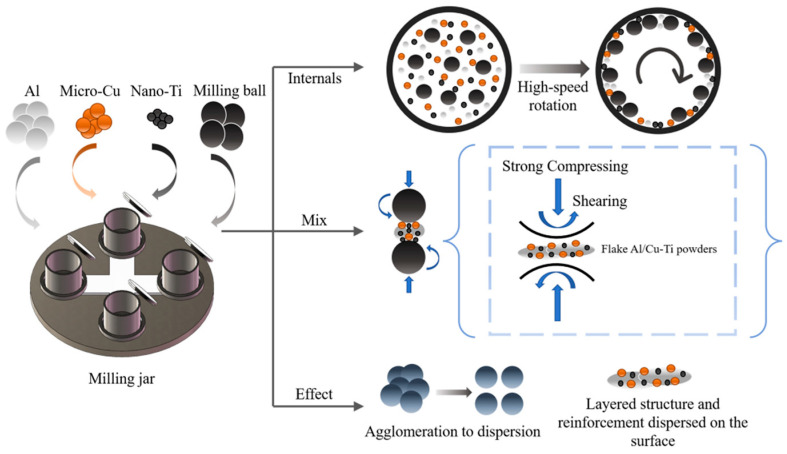
Schematic diagram of the evolution of BM mixed powder.

The micro-nano dual-scale hybrid particles contained 5 wt%Cu and different contents of nano Ti (0, 5, 10, 15 wt%). Meanwhile, after determining the optimal strength effect, large-particle Al (10 wt%, 15 wt%) was further added to balance plasticity. As a control, AMCs without reinforcement or with single-type particles were also prepared under the same conditions. All samples in the study are presented in Table 2. To achieve uniform distribution of reinforcement and refinement of particles, the powder was ball-milled in a planetary ball mill at 200 rpm for 6 h. Prior to ball milling, zirconia balls with diameters of 3 mm and 6 mm (in a quantity ratio of 1:3) were placed into an agate ball mill jar first. Subsequently, various types of powders with different contents were loaded into the jar under an argon atmosphere. Among the process parameters, the total mass ratio of balls to powders was controlled at 10:1, and 1 wt% stearic acid was added as a process control agent to prevent excessive cold welding of the powder. The evolution diagram of the BM mixed powder is shown in Figure 1. The parameters of BM were derived from the previous study [27]. Subsequently, the milled powders were filled into a groove with the dimension 4 mm in width and 3 mm in depth. The groove was cut at the center and along the rolling direction of the sheets. Furthermore, a cover plate was used to prevent the powders from splashing during processing. After filling the powders, FSP was conducted on the groove area using a pin tool with 3 passes. The rotation speed, traverse speed, and tilt angle of the FSP tool were kept constant at 1200 rpm, 40 mm/min, and 3°, respectively [3].

The prepared composites were subjected to grinding, polishing, and subsequent etching using Keller’s reagent (95 mL H_2_O, 2.5 mL HNO_3_, 1.5 mL HCl, 1 mL HF) for 15 s. The microstructure of the SZ was examined using an optical microscope (OM) (Zeiss, Oberkochen, Germany) and a field emission scanning electron microscope (FESEM) (Zeiss, Oberkochen, Germany) coupled with energy-dispersive X-ray spectroscopy (EDS) (Oxford Instruments, Concord, MA, USA) for simultaneous analysis. Phase identification of the milled powder and FSPed samples was conducted via X-ray diffraction (XRD) (Rigaku, Wilmington, MA, USA). The grain size measurement of the sample was accomplished using the ImageJ image analysis software (v.1.53b). When measuring, three regions were selected from each sample for sampling, and ≥100 grains were counted in each region. Subsequently, the statistically obtained grains underwent data processing, and origin software (v. 9.9.0.225) was used to draw the grain size distribution map. The discussion of some sub-micron mechanism scales in this paper is derived from a comprehensive analysis of existing experimental characterization methods and previous studies.

**Figure 2:**
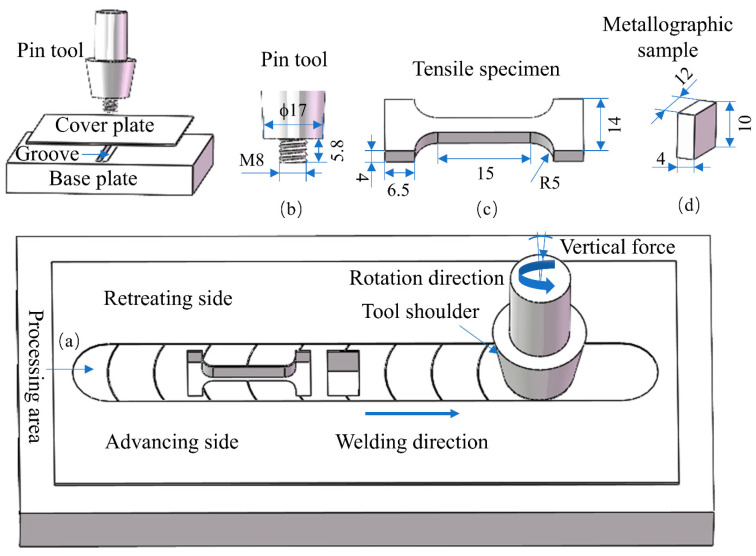
Schematic of FSP accomplishment: (**a**) processing area, (**b**) pin tool, (**c**) tensile specimen, and (**d**) metallographic sample.

In addition, the mechanical properties, including the stress–strain curves and hardness, were also tested in this experiment. Tensile specimens with a dog bone shape were extracted along the processing direction from the SZ using electro-discharge machining and evaluated on an electronic universal testing machine (INSTRON, Norwood, MA, USA) at a strain rate of 1 × 10^−3^ s^−1^ under ambient conditions. Vickers hardness was assessed using a microhardness tester (HUAYIN, Laizhou, China) with a 100 gf load applied for 10 s. These mechanical property assessments were conducted ten times, and the averaged results were documented. Specimen geometry is illustrated in Figure 2.

## 3. Results

### 3.1. Morphology and Phase Analysis of the Milled Powders

**Figure 3:**
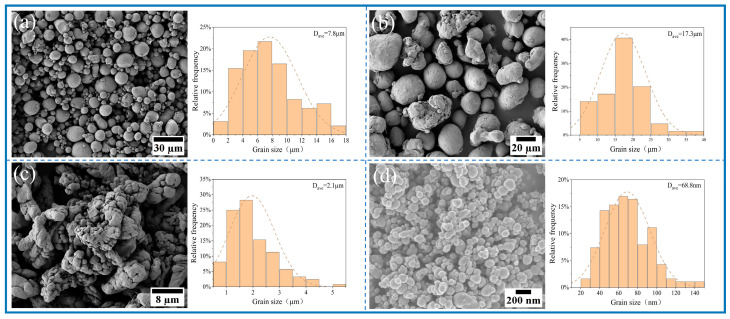
SEM image and particle size distributions of (**a**) fine-particle Al (**b**) coarse-particle Al, (**c**) micro Cu, and (**d**) nano Ti.

**Figure 4:**
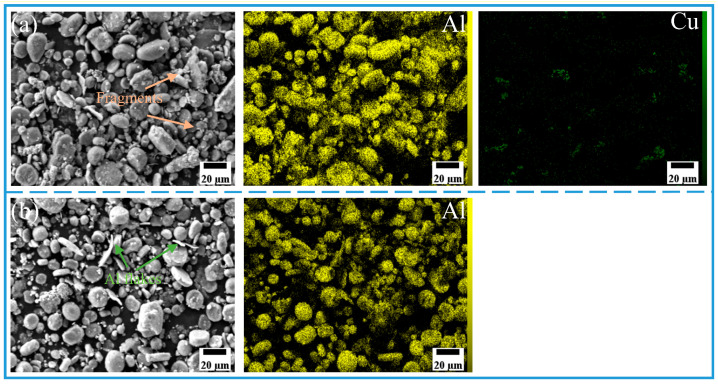
SEM image and EDS mapping analysis of milled powder: (**a**) Al_10_-5Cu, (**b**) 100%Al_10_.

**Figure 5:**
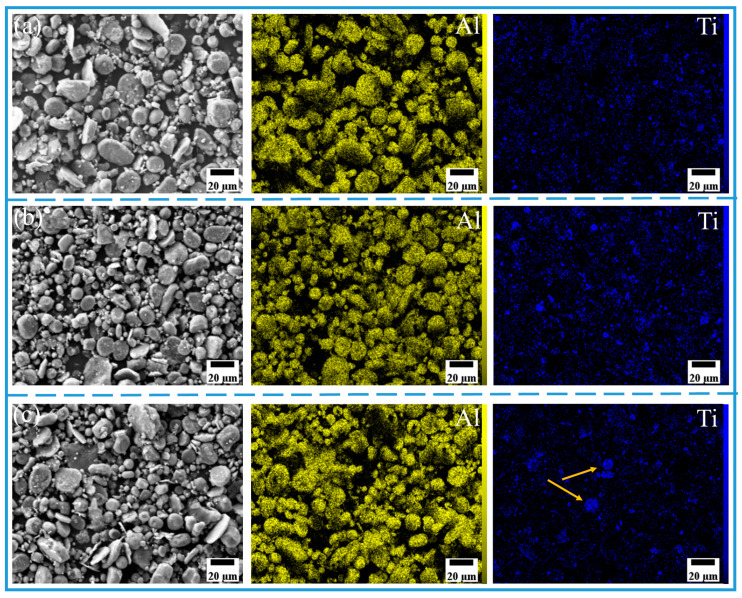
SEM image and EDS mapping analysis of milled powder: (**a**) Al_10_-5Ti, (**b**) Al_10_-10Ti, and (**c**) Al_10_-15Ti.

**Figure 6:**
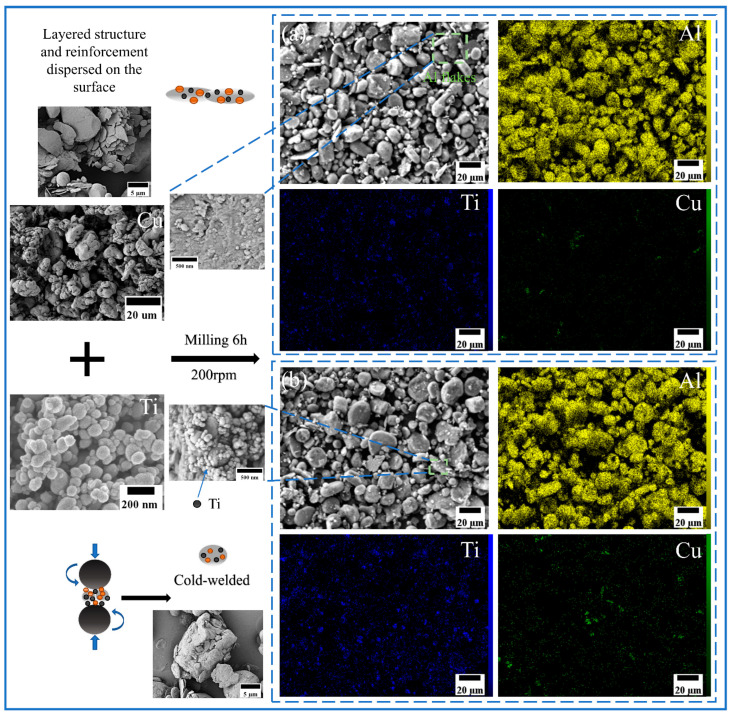
SEM image and EDS mapping analysis of milled powder: (**a**) Al_10_-5Cu-5Ti, (**b**) Al_10_-5Cu-10Ti.

**Figure 7:**
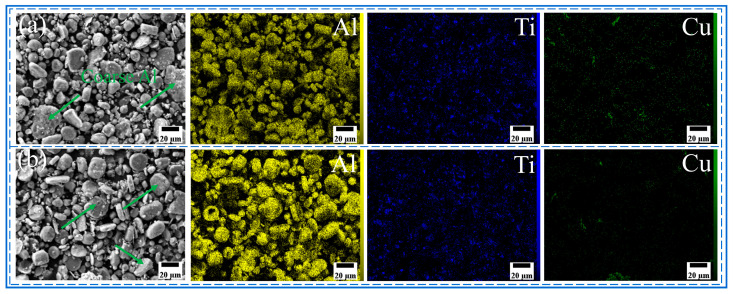
SEM image and EDS mapping analysis of milled powder: (**a**) Al_10_-5Cu-10Ti-10Al_20_, (**b**) Al_10_-5Cu-10Ti-15Al_20_.

The morphology and size distribution of the raw material powder are shown in Figure 3. Figure 4, Figure 5, Figure 6 and Figure 7 show the morphology of the milled powders after 6 h combined with EDS analysis. It could be seen that the spherical Al became flattened and fragmented into smaller pieces after BM, which was caused by the collisions between the balls. The treatment significantly enhanced the specific surface area of the aluminum matrix, thereby improving its interfacial bonding with reinforcement particles. EDS analysis confirmed that the BM process effectively achieved more uniform dispersion of both micron-scale Cu and nano-scale Ti reinforcing particles. However, EDS analysis of the 15 wt% Ti sample revealed localized particle agglomeration (orange arrow in Figure 5c). Furthermore, small bright particles were found on the surface of the flake (green dashed box in Figure 6), which were inferred to be nano Ti combined with flake Al.

According to the study, the radial compressive force and tangential shear force generated by BM collisions play crucial roles in the refinement of Al powder particle size and the dispersion of reinforcement. The spherical Al was directly compressed by the radial compressive force, which underwent plastic deformation and gradually transitioned to a flattened state [29]. During continued compaction of balls, flake Al was cold-welded into larger lamellar structures, which were subsequently fragmented into smaller particles. After the repeated “cold welding-fragmentation” process, the particle size reached a dynamic equilibrium to achieve the reduction of particle size. Compared to the milled powder without reinforcement, the addition of reinforcement significantly promoted the refinement of spherical Al powders during BM. The reinforcements usually serve as hard media to improve the energy transfer efficiency during BM. They act as chisels to enhance the impact of the compressive force, which promote the plastic deformation and fragmentation of the flake Al [30]. Therefore, the addition of reinforcements effectively suppresses the tendency for cold welding to promote the refinement of Al and the uniformity of morphology.

When the grinding balls collide with the powder, the generated shear force creates sliding friction on the particle surfaces [30]. The particle could be dispersed and adhered on the surfaces of larger flake Al effectively. Meanwhile, the large surface area of the flake Al was conductive to the adhesion of reinforcement. Under the action of compressive force, the reinforcement bonded more strongly with the matrix, which is conducive to in situ reactions during subsequent multipass FSP.

**Figure 8:**
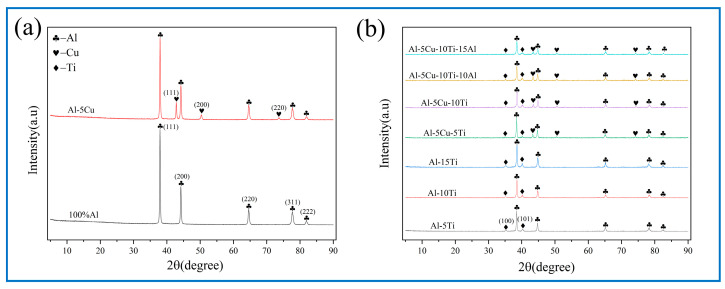
XRD of milled powder after BM: (**a**) free of Ti, (**b**) containing Ti.

Figure 8 shows the XRD results of milled powder, which indicates that the diffraction peaks correspond exclusively to Al, Cu, and Ti. The diffraction peaks of Al were observed at 2θ angles of 38.5°, 44.7°, 65.1°, 78.2°, and 82.5°. Meanwhile, the characteristic peaks of Cu were shown at 43.1°, 50.3°, and 74.1° [31,32], while Ti was detected at 35.3° and 40.3° [2,33,34]. Notably, no new phases were detected in all milled powders after BM, which suggests that no in situ reaction occurred.

### 3.2. Microstructure and Phase Analysis of AMCs

**Figure 9:**
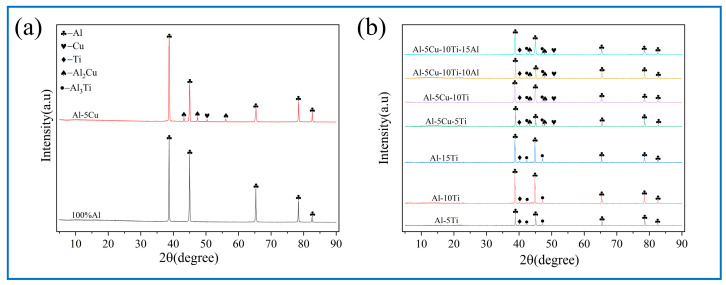
XRD pattern of all FSPed samples: (**a**) free of Ti, (**b**) containing Ti.

Crystalline phase evolution of the treated AMCs was systematically characterized via XRD. This analysis confirmed the structural integrity and phase composition of the material after FSP, aligning with the expected crystallographic transformations under the applied processing conditions. All XRD-detected characteristic peaks exhibited precise alignment (as shown in Figure 9), which has been documented in prior studies [2,31,32,33,34].

Phase identification via XRD analysis confirmed the presence of Al_2_Cu and Al_3_Ti intermetallic compounds within the Cu/Ti-reinforced AMCs, demonstrating the feasibility of in situ synthesis of these strengthening phases through a multi-pass FSP strategy. The observed enhancement in XRD peak intensity of intermetallic compounds and simultaneous attenuation of corresponding metallic peaks can be attributed to in situ reaction mechanisms. On the one hand, the low solid solubility of Cu/Ti in Al leads to the precipitation of intermetallic compounds phases when Cu/Ti concentrations exceed critical solubility thresholds. On the other hand, FSP of Al alloys generates SZ temperatures exceeding 400 °C. Prior studies have confirmed that Al_3_Ti and Al_2_Cu intermetallic nucleation initiates around 400 °C under equilibrium solid-state reaction conditions [35,36]. The plastic deformation of the material surrounding the pin during FSP contributes to heat generation, with an elevated tool rotation speed to traverse speed ratio inducing higher thermal input to AMCs. This increased thermal exposure facilitates the formation of intermetallic compounds, as reported in prior investigations [37]. Moreover, the BM-induced homogeneous Cu/Ti distribution across the Al foil surface enables uniform nucleation of intermetallic phases during interfacial reactions.

**Figure 10:**
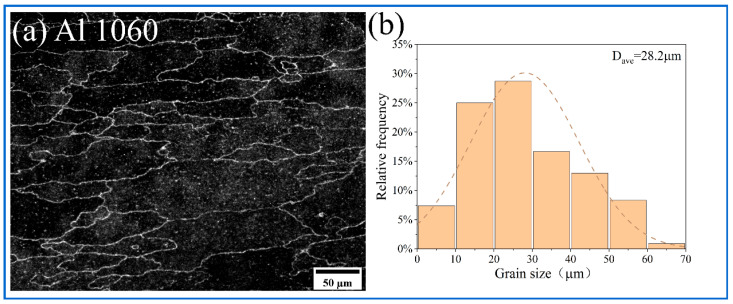
(**a**) Metallographic structure; (**b**) grain size distribution of Al 1060.

**Figure 11:**
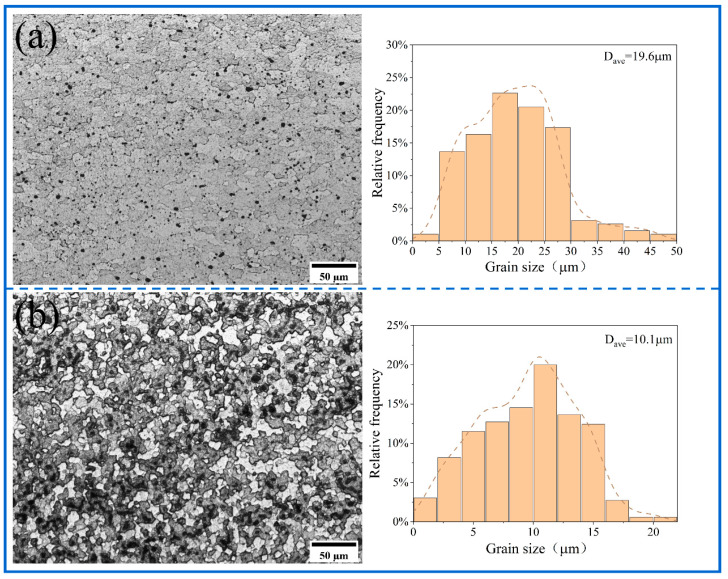
The optical microstructure of SZ and grain size distribution of (**a**) 100% Al_10_, (**b**) Al_10_-5Cu.

**Figure 12:**
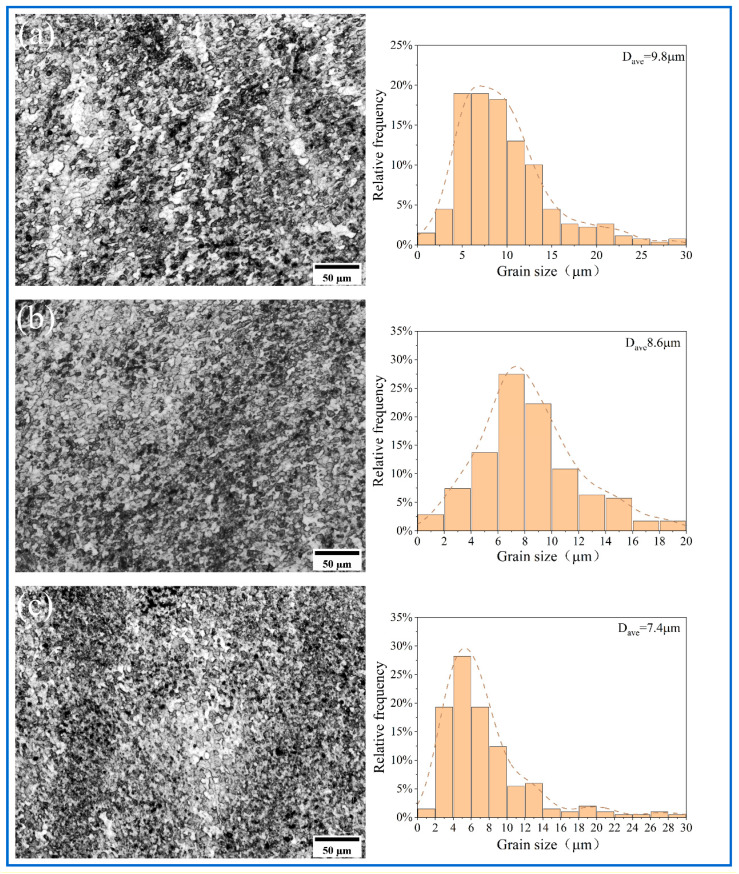
The optical microstructure of SZ and grain size distribution of (**a**) Al_10_-5Ti, (**b**) Al_10_-10Ti, (**c**) Al_10_-15Ti.

**Figure 13:**
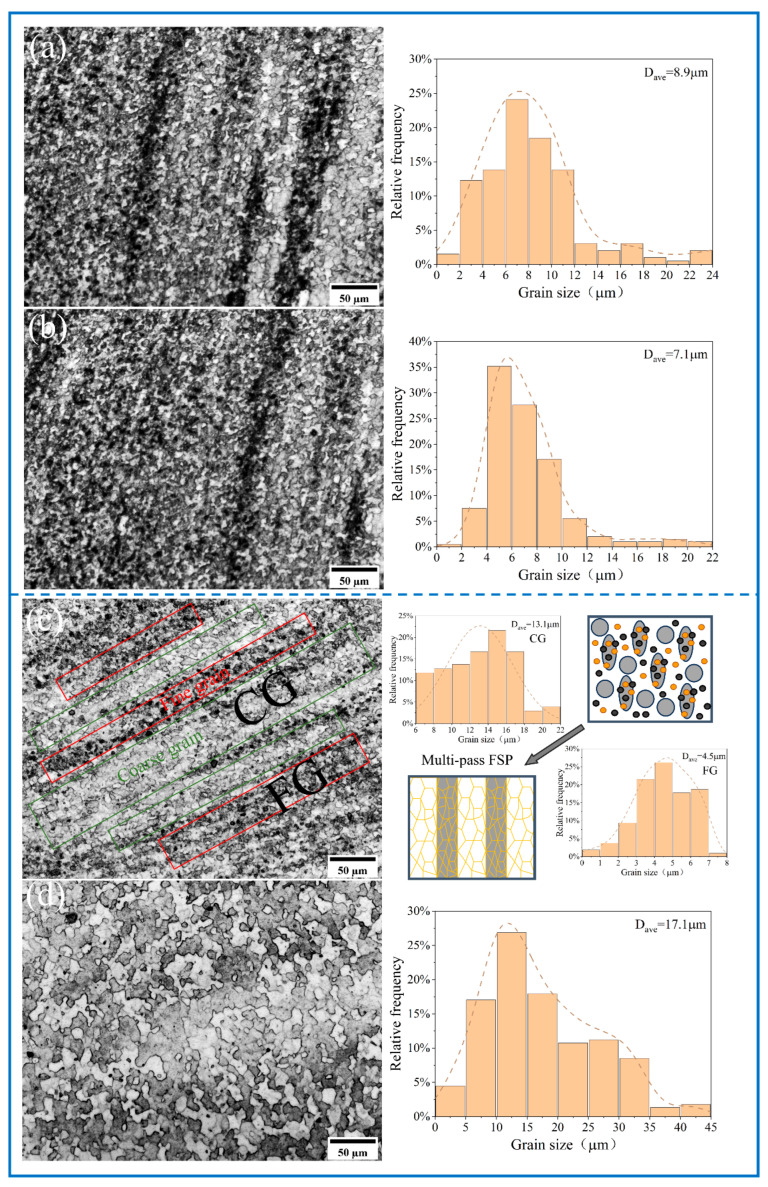
The optical microstructure of SZ and grain size distribution of (**a**) Al_10_-5Cu-5Ti, (**b**) Al_10_-5Cu-10Ti, (**c**) Al_10_-5Cu-10Ti-10Al_20_, and (**d**) Al_10_-5Cu-10Ti-15Al_20_.

The metallographic structure of the substrate is shown in Figure 10. Figure 11, Figure 12 and Figure 13 show the microstructure of all samples fabricated by multi-pass FSP. Combined with the base metal, all samples showed a significant grain size reduction. Grain refinement caused by multipass FSP was achieved through DRX, which was influenced by SPD and heat input [37]. The high-speed rotation of the threaded tool generated significant shear stress and a high density of dislocations, which provided the driving force for recrystallization. Additionally, the heat input from friction between the tool and material induced DRX [38,39]. This process transforms the original coarse grains into fine equiaxed grains. The newly formed nuclei are further refined under continuous deformation and thermal cycling, resulting in a significant reduction in average grain size to the micrometer level. The synergistic effects of plastic deformation and thermal input drive grain refinement through dislocation-mediated nucleation and grain boundary migration [40].

Compared with the unreinforced AMCs, the Al_10_-5Cu alloy exhibited an average grain size of 10.1 μm. The inconsistent grain refinement observed in localized regions was attributed to inconsistent particle distribution within the matrix. In contrast, the Al_10_-5Ti composite displayed a coarser average grain size of 9.8 μm, suggesting limited efficacy of 5 wt% Ti additions in suppressing grain growth. The experimental results demonstrate that elevating the Ti content to 10 wt% and 15 wt% leads to substantial microstructure refinement, with the average grain size decreasing to 8.6 μm and 7.4 μm, respectively. Nevertheless, grain coarsening phenomena are observed in certain agglomerated zones, indicating inhomogeneous distribution of the refining effect. AMCs reinforced with hybrid micron-scale Cu and nano-scale Ti particles demonstrate superior grain refinement, achieving reduced average grain sizes of 8.9 μm (Al_10_-5Cu-5Ti) and 7.1 μm (Al_10_-5Cu-10Ti), respectively. Localized strain concentration zones are induced within the matrix due to the incorporation of reinforcing particles, generating regions with elevated dislocation densities. The resultant dislocation accumulation in these zones serves as a critical thermodynamic driver for DRX, thereby accelerating microstructural refinement through enhanced nucleation of equiaxed grains [41,42]. Furthermore, the reinforcement particles inhibits grain growth by impeding grain boundary migration and dislocation motion, which can restrict the growth of new grains during DRX. The preferential efficacy of nano-Ti incorporation in grain refinement mechanisms relative to micron-scale Cu counterparts is attributed to the nanoscale dimensions conferring elevated specific surface areas, which critically enhance interfacial energy-driven pinning effects and promote heterogeneous nucleation site density [17,43]. Thus, nano-Ti results in superior grain refinement compared to micron-sized particles.

The incorporation of coarse Al powder into the Al_10_-5Cu-10Ti system led to significant microstructural heterogeneity. The micrograph of the Al_10_-5Cu-10Ti-10Al_20_ composite material shows the characteristics of heterogeneous structure, with a large grain area size of 13.1 μm and a small grain area size of 4.5 μm. With further Al addition (15 wt.%), the Al_10_-5Cu-10Ti-15Al_20_ sample demonstrated more pronounced grain coarsening (17.1 μm). This phenomenon can be attributed to the agglomeration of oversized Al particles at interfaces, which significantly weakened their grain boundary pinning effect. The non-uniform distribution of these obstacles consequently reduced the local resistance to grain boundary migration.

Compared with individual micron-sized Cu or nano-sized Ti reinforcements, the hybrid reinforcement system composed of micron-scale Cu and nano-scale Ti particles demonstrates superior optimization effects on both microstructure refinement and grain size control in AMCs. Ti nanoparticle-dense zones exhibited refined microstructures attributable to enhanced Orowan strengthening efficacy, while microcopper-concentrated domains were typified by coarser crystallographic structure features. Nano-sized Ti enhances the grain refinement effect of micron-sized Cu by leveraging its unique advantages. Micron-sized Cu particles, as the primary reinforcing phase, form a macroscopic skeleton structure, restricting the coarsening of the matrix grains through geometric constraint effects and providing structural support. Nano-sized Ti particles, due to their high surface energy and fine size, further inhibit grain growth and promote matrix grain refinement [17]. The synergistic effects of the two not only improve the dispersion uniformity of the reinforcement phase but also effectively reduce the grain size through the pinning effect and heterogeneous nucleation mechanism.

The incorporation of coarse Al, while leading to increased grain size, significantly enhances the deformability during FSP. These CGs elongate preferentially along the extrusion direction, alternating with the FG zone composed of reinforcing particles and eventually forming a heterogeneous structure. This unique microstructure configuration endows the composite material with graded deformation capability [27].

To further evaluate the microstructure, the relative density values were measured to assess the compactness of the samples (as shown in Table 3). Experiments indicate that after multi-pass FSP, the plastic flow and DRX of the material are conducive to improving its internal structure and eliminating defects. Meanwhile, the content of reinforcement particles is also closely related to the magnitude of relative density. For samples containing only 5 wt% Cu or Ti, the improvement in structure is relatively limited due to their low content. However, nano-sized Ti, with its higher specific surface area, can be uniformly dispersed, which is beneficial for increasing the overall density of the material. In contrast, the relative density of AMCs with 15 wt% Ti added decreases significantly, which may be attributed to the agglomeration of nano-sized Ti particles. For AMCs reinforced with hybrid particles, the relative density has been significantly improved. The presence of micro-sized Cu facilitates the dispersion of nano-sized Ti, while nano-sized Ti further complements the role of micro-sized Cu to optimize the microstructure [44]. The variation trend of relative density is consistent with that reported in previous studies [45,46]. Furthermore, intermetallic compounds formed in situ also contribute to microstructure optimization, thereby reducing porosity and increasing relative density. In summary, the improvement in relative density reflects the changes in the microstructure. A higher relative density also indicates that the stir zone is in an ideally compact state.

**Figure 14:**
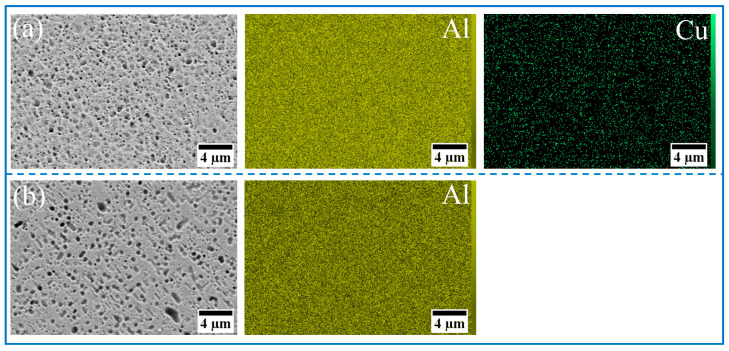
SEM image and EDS mapping analysis of the SZ of AMCs: (**a**) 100%Al_10_, (**b**) Al_10_-5Cu.

**Figure 15:**
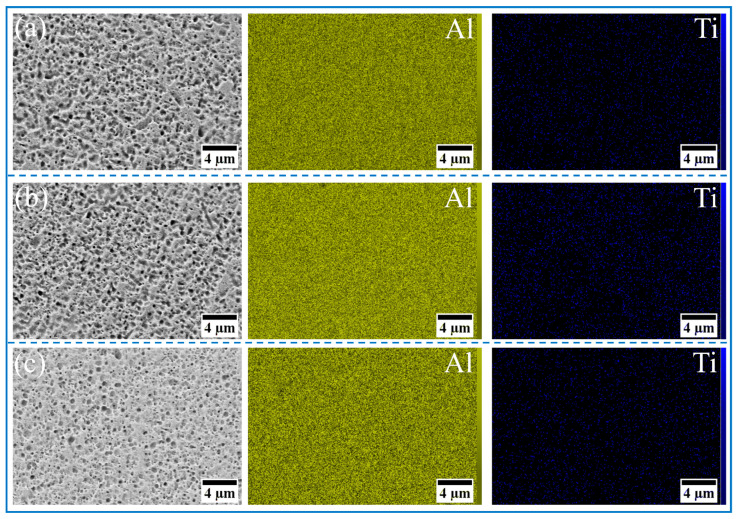
SEM image and EDS mapping analysis of the SZ of AMCs: (**a**) Al_10_-5Ti, (**b**) Al_10_-10Ti, and (**c**) Al_10_-15Ti.

**Figure 16:**
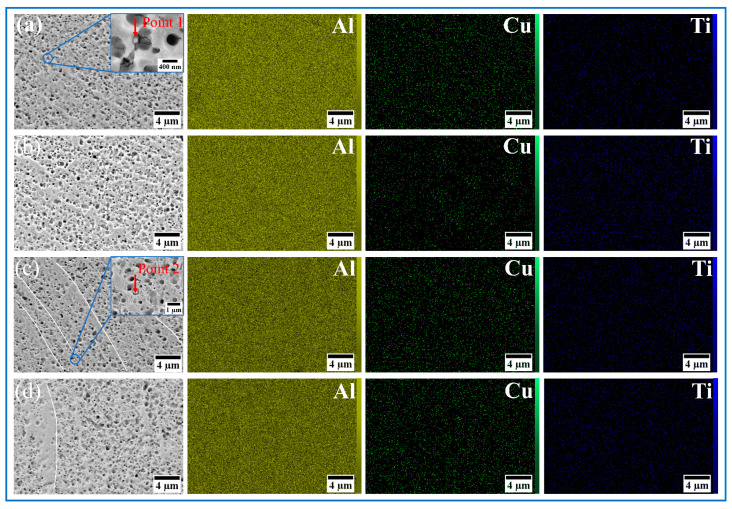
SEM image and EDS mapping analysis of the SZ of AMCs: (**a**) Al_10_-5Cu-5Ti, (**b**) Al_10_-5Cu-10Ti, (**c**) Al_10_-5Cu-10Ti-10Al_20_, and (**d**) Al_10_-5Cu-10Ti-15Al_20_.

SEM characterization of the SZ in AMCs is presented in Figure 14, Figure 15 and Figure 16, with no macroscale discontinuities (e.g., tunneling defects or crack formation) being identified during three-pass FSP. The incorporation of reinforcement particles leads to a significant reduction of deep pores in the AMC microstructure, with the surface predominantly exhibiting small and shallow dimples. An obvious banded structure was found in the Al10-5Cu-10Ti-10Al20 composite material, which might be related to the heterogeneous architecture (Figure 16c, white line). During FSP, coarse Al powders elongate along the extrusion direction to form elongated CG bands due to their superior deformability. This results in a multi-scale heterogeneous architecture, alternating with refined regions containing micron-sized Cu and nano-Ti reinforcing particles. Elemental composition and spatial distribution of metallic reinforcements were characterized through EDS analysis. Homogeneous dispersion of Ti and Cu was achieved via the synergistic interaction between the BM and multi-pass FSP, facilitating intermetallic compound formation (e.g., Al_3_Ti and Al_2_Cu). Compositional verification of these regions was performed through localized spot scanning.

**Figure 17:**
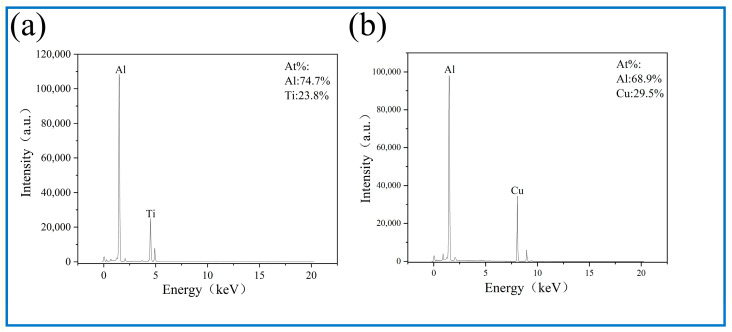
EDS spectrum corresponding to enlarged image in Figure 16: (**a**) point 1, (**b**) point 2.

A sandwich architecture was identified in the AMCs within the Al_10_-5Cu-10Ti-10Al_20_ (Figure 16c). EDS point analysis confirmed Cu dominance in bright micrometer-scale particulate phases, while the gray interfacial layer was determined as Al_2_Cu based on stoichiometric atomic ratios. Cu-Al interdiffusion during multi-pass FSP was observed to facilitate nanoscale Al_2_Cu precipitation. Progressive darkening of laminar structures adjacent to the substrate was identified, consistent with prior reports [4]. Lamellar intermetallic phases were also identified at particulate–matrix interfaces in Al_10_-5Cu-5Ti AMCs (Figure 16a), characterized by bright particulate phases encircled by architectured gray lamellar regions [33]. In point 1 (Figure 17a), Al/Ti atomic ratios of 3.14 were measured, indicative of Al_3_Ti formation. XRD analyses (Figure 9) confirmed the coexistence of Al_3_Ti and Al_2_Cu intermetallic phases within post-heat-treatment processed zones, exhibiting strong consistency with EDS compositional data.

Integrated with EDS point-scan data, the in situ reaction mechanism of reinforcement particulates was characterized as follows: lamellar intermetallic phases formed at particulate–substrate interfaces undergo spallation due to SPD induced by FSP [47]. Exposed particulates subsequently participate in continued interfacial reactions, with cyclic depletion occurring progressively during material flow. A minor fraction of reinforcement particulates remains retained within the matrix, attributed to limited processing duration.

The synergistic interaction between nano- and microparticles enhances dispersion homogeneity in particle-reinforced AMCs. Homogeneous nanoparticle dispersion is promoted by the presence of microparticles, which serve as preferential dispersion sites during FSP. These microparticles facilitate nanoparticle anchoring through interfacial interactions, enabling preferential spatial distribution within the matrix [48]. The incorporation of large-sized Al powders facilitates void filling and particle distribution optimization, thereby enhancing load transfer efficiency and mitigating stress concentration, which consequently improves both strength and plasticity [49,50].

### 3.3. Mechanical Properties

**Figure 18:**
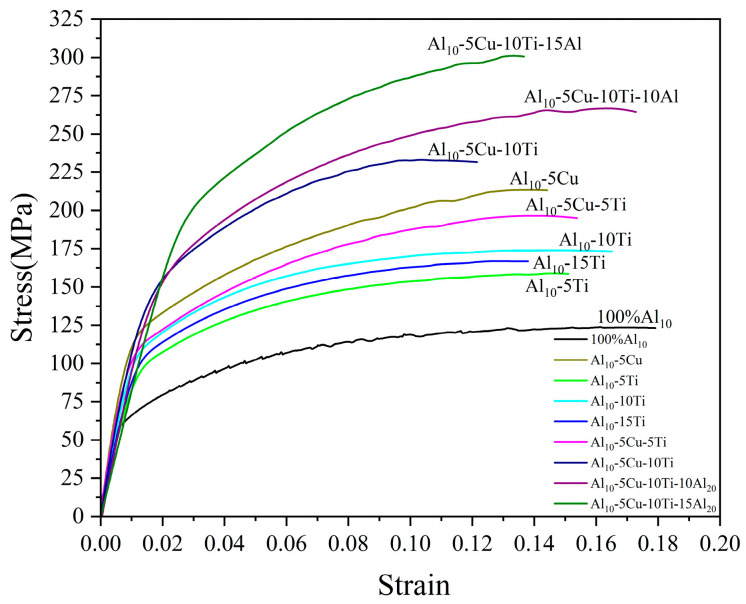
The representative tensile curve of AMCs.

The mechanical properties of the fabricated AMCs were experimentally evaluated, with the corresponding stress–strain relationships presented in Figure 18. Significant enhancements in mechanical properties were observed across all AMCs when compared to the base matrix material. Detailed comparative data quantifying these property improvements have been systematically compiled in Table 4. The unreinforced AMCs exhibited measured UST and elongation values of 124 MPa and 17.0%, respectively. The observed mechanical properties enhancement could be attributed to grain refinement and microstructural homogenization achieved through DRX initiated during FSP. [51]. The Al_10_-5Cu alloy exhibited a measured UST of 214 MPa along with 14.0% elongation. Significant mechanical strengthening was achieved through controlled Cu additions, while concomitant ductility reduction was observed. Enhanced load-bearing capacity was conferred on AMCs through the incorporation of micron-scale Cu dispersoids, where in situ interfacial reactions generated Al_2_Cu intermetallics that reinforced interfacial cohesion, thereby achieving significant strength enhancement. However, plasticity deterioration was induced by localized stress concentrations originating from coarse Cu particulates. Furthermore, crack nucleation susceptibility was elevated by the inherent brittleness of phases formed in situ [31,52]. UTS values for composites containing 5 wt%, 10 wt%, and 15 wt% Ti additions are measured at 159 MPa, 174 MPa, and 167 MPa, respectively, while corresponding elongations reach 14.6%, 15.7%, and 12.8%. Performance degradation in the 15 wt% Ti-AMCs is attributed to insufficient interfacial adhesion between particle agglomerates and the matrix, which significantly compromises load transfer efficiency and accelerates failure mechanisms, ultimately reducing both strength and ductility. In contrast, hybrid composites incorporating both Cu and Ti (Al_10_-5Cu-(5-10)Ti system) exhibit superior mechanical characteristics, achieving UTS values of 197 MPa and 233 MPa with elongations of 14.1% and 10.3%, respectively.

The mechanical properties of AMCs were significantly enhanced through the incorporation of coarse Al particles, with the Al_10_-5Cu-10Ti-10Al_20_ and Al_10_-5Cu-10Ti-15Al_20_ composites exhibiting tensile strengths of 267 MPa and 301MPa, coupled with elongations of 16.7% and 13.3%, respectively. While the Al_10_-5Cu-10Ti-15Al_20_ composite demonstrated a remarkable 230.8% strength increase over the matrix alloy, its elevated coarse Al content (15 wt%) led to particle clustering and inhomogeneous reinforcement distribution, creating stress concentration sites that served as microcrack initiation points and consequently reduced ductility. In contrast, the Al_10_-5Cu-10Ti-10Al_20_ composite achieved a synergistic improvement in both strength and plasticity, as the optimal 10 wt% coarse Al addition facilitated the development of a bimodal grain structure consisting of CG and FG zones. This heterostructure effectively accommodated strain through coordinated deformation mechanisms, thereby enhancing the overall mechanical performance.

The concurrent enhancement in tensile strength and retention of elongation is attributed to the synergistic interplay of grain refinement and multi-scale strengthening mechanisms. Grain refinement governs strengthening via the Hall–Petch relationship, simultaneously increasing grain boundary density to promote homogeneous plastic deformation and suppress crack propagation, thereby preserving ductility. This is augmented by three complementary mechanisms: (i) Orowan strengthening by nano-Ti dispersoids; (ii) effective load transfer mediated by intermetallic compounds formed in situ (Al_3_Ti/Al_2_Cu); and (iii) thermal mismatch strengthening at the Al-matrix/Ti-Cu reinforcement interfaces.

The CG zones function as a soft phase, preserving substantial plasticity. These regions serve as “plasticity reservoirs” that promote homogeneous strain distribution through back stress hardening effects, thus delaying localized necking. Concurrently, the hetero-interfacial domains maintain sustained work hardening capacity via continuous multiplication of geometrically necessary dislocations [49,53]. This coordinated mechanism achieves simultaneous enhancement of both strength and ductility.

**Table 4:** The mechanical properties of AMCs in this experiment and other literature.

Material	Hardness/HV	UTS/MPa	El/%
Al1060	30	91	15.2
100%Al_10_	45 ± 2	124 ± 2	17.0 ± 0.7
Al_10_-5Cu	74 ± 3	214 ± 2	14.0 ± 0.4
Al_10_-5Ti	54 ± 2	159 ± 3	14.6 ± 0.5
Al_10_-10Ti	59 ± 2	174 ± 3	15.7 ± 0.2
Al_10_-15Ti	56 ± 2	167 ± 3	12.8 ± 0.2
Al_10_-5Cu-5Ti	68 ± 3	197 ± 2	14.1 ± 0.5
Al_10_-5Cu-10Ti	89 ± 3	233 ± 2	10.3 ± 0.4
Al_10_-5Cu-10Ti-10Al_20_	98 ± 3	267 ± 6	16.7 ± 0.3
Al_10_-5Cu-10Ti-15Al_20_	103 ± 4	301 ± 5	13.3 ± 0.4
AA1050/Fe [54]	73	160	20
AA1050/Fe-Cu [55]	73	207	1/10 of the substrate
Al1060/Ni [43]	66	184	20.1
AA5083/SiC [56]	\	335	7.4
AA2014-AA2024/TiO_2_ [57]	90	259	\

**Figure 19:**
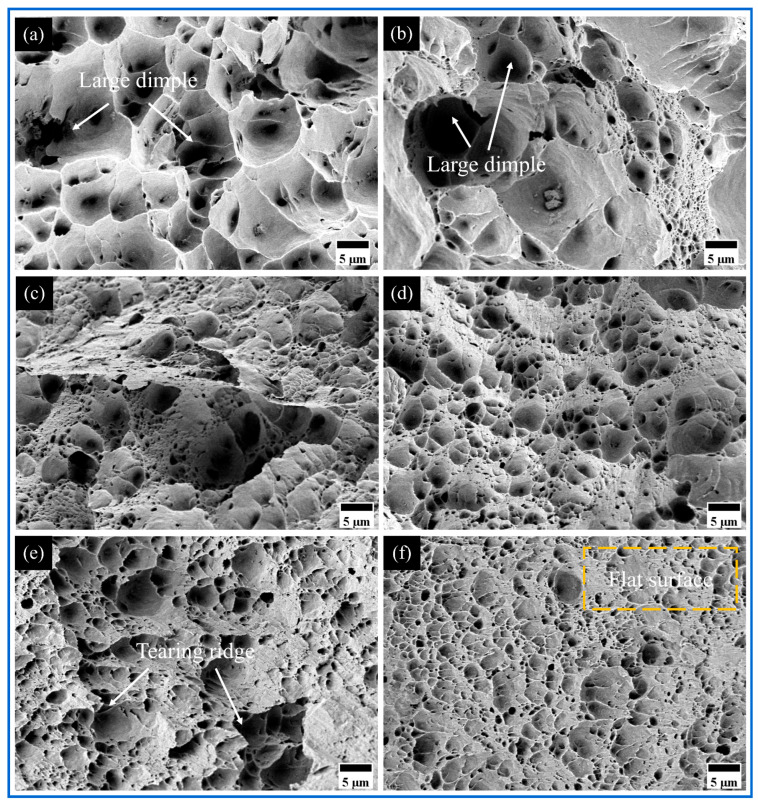
The fracture morphology of (**a**) Al 1060, (**b**) 100%Al_10_, (**c**) Al_10_-5Cu, (**d**) Al_10_-5Ti, (**e**) Al_10_-10Ti, and (**f**) Al_10_-15Ti.

**Figure 20:**
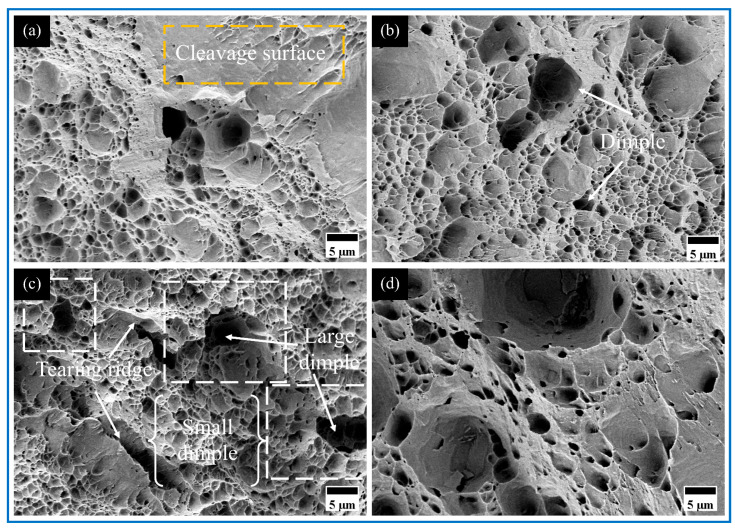
The fracture morphology of (**a**) Al_10_-5Cu-5Ti, (**b**) Al_10_-5Cu-10Ti, (**c**) Al_10_-5Cu-10Ti-10Al_20_, and (**d**) Al_10_-5Cu-10Ti-15Al_20_.

Fractographic analysis of AMCs (Figure 19 and Figure 20) revealed surface morphologies consistent with corresponding stress–strain responses. Uniform dimple structures characteristic of ductile failure mechanisms were identified across all specimens. Progressive dimensional reduction of primary dimples was observed with reinforcement incorporation, accompanied by secondary dimple formation showing uniform dispersion surrounding primary cavities. This microstructural evolution confirms effective interfacial bonding via matrix–particle cohesion, demonstrating enhanced deformation characteristics comparable to the matrix ductility.

The fracture surfaces of micro-Cu reinforced Al_10_-5Cu AMCs exhibit underdeveloped and inhomogeneously distributed dimple structures. This diminished ductility primarily stems from the formation of brittle Al_2_Cu intermetallic compounds and insufficient bonding strength at particle–matrix interfaces [52]. Incorporation of nano-Ti particles induces refined dimple morphology characterized by reduced size, increased depth, and uniform spatial distribution across the fracture surface. Such microstructural evolution confirms effective interfacial bonding between reinforcement and matrix, correlating with enhanced tensile strength and ductility in modified alloy systems. In Al_10_-5Cu-5Ti and Al_10_-5Cu-10Ti AMCs (Figure 20), a progressive diminution of coarse shallow dimples occurs alongside elevated dimple density. Nevertheless, the concomitant emergence of planar fracture facets corresponds to reduced plasticity.

Experimental results demonstrate that 10 wt% coarse Al particles represent the optimal reinforcement content, beyond which excessive additions (e.g., 15 wt% Ti) induce particle agglomeration, leading to deteriorated dimple structures and the formation of flat fracture surfaces with significantly reduced plasticity. In Al_10_-5Cu-10Ti-10Al_20_ AMCs, the incorporation of coarse Al particles promotes deeper dimple formation, accompanied by densely distributed micron-scale satellite dimples around primary dimples (white box in Figure 20c), highlighting the synergistic effect of multi-scale reinforcement. The fracture surfaces exhibit increased tear ridge density with sharper contours, indicating enhanced resistance to plastic deformation during fracture and confirming the strengthening-toughening advantages of the dual-scale architecture. This microstructural evolution originates from the elevated microvoid nucleation site density induced by higher reinforcement particle concentration [58]. The Al_3_Ti phase formed in situ strengthens interfacial bonding, effectively impeding and deflecting crack propagation paths during deformation. Furthermore, coarse/fine grain boundaries and particle-matrix interfaces serve as potent dislocation sources, continuously emitting new dislocations while hindering their glide [59]. This mechanism leads to high strain gradient formation at deformation band intersections, ultimately manifesting as high complexity of tear ridges and achieving balanced improvements in both strength and ductility.

**Figure 21:**
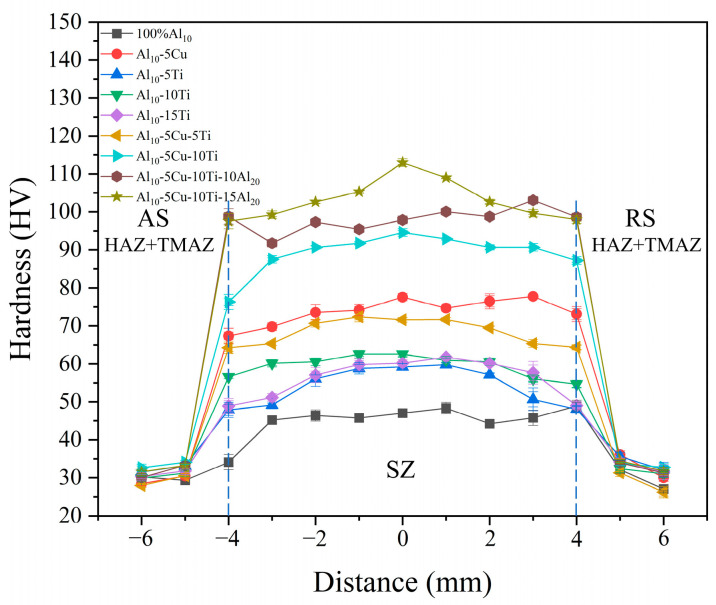
The microhardness profile of AMCs.

The Vickers microhardness profiles of AMCs are presented in Figure 21. Hardness measurements were conducted symmetrically across weld zones at 1 mm intervals, spanning ±6 mm from the weld centerline. A uniform hardness enhancement was observed throughout all AMCs weld regions compared to base material values. Notably, hybrid-reinforced AMCs exhibit superior hardness improvement compared to single-particle reinforced counterparts. The Al_10_-5Cu-10Ti-10Al_20_ and Al_10_-5Cu-10Ti-15Al_20_ composites achieve hardness values of 98 HV and 103 HV, respectively, representing 226.7% and 243.3% increases over the base material.

The enhanced hardness of AMC samples was attributed to three primary mechanisms. First, grain refinement strengthening was achieved through DRX and thermomechanical processing, which reduced the average grain size of the matrix in alignment with the Hall–Petch relationship [60]. Second, Al_2_Cu and Al_3_Ti phases formed in situ enhanced the matrix via the Orowan mechanism, where dislocation loops around these precipitates improved deformation resistance and hardness [47,61]. Third, thermal and mechanical mismatches between reinforcement particles and the matrix during FSP generated high-density dislocation networks due to differences in thermal expansion coefficients among Ti, Cu, and Al. These dislocations further impeded dislocation motion through strain field interactions [41].

Additionally, multi-pass FSP contributed to hardness improvement through localized heating and mechanical stirring, which promoted SPD. This process simultaneously refined grain sizes and homogenized the distribution of reinforcing phases. The synergistic effects of grain boundary strengthening, precipitation hardening, and dislocation interactions collectively enhanced hardness, particularly in the SZ [62].

## 4. Conclusions

This investigation successfully addressed the strength–ductility trade-off in AMCs through a synergistic design incorporating dual-scale hybrid reinforcements (micro-Cu/nano-Ti) and a bimodal Al matrix, which were fabricated via combined BM and multi-pass FSP techniques. The principal findings demonstrate:

1. The homogeneous dispersion of micron-scale Cu particles and nano-Ti particles was achieved through iterative cold welding and fracturing cycles, concurrently refining the Al powder morphology. Subsequent multi-pass FSP effectively facilitated the in situ formation of intermetallic compounds while enabling the architectural design of heterogeneous microstructures.
2. The composite materials exhibited significant grain refinement due to the synergistic effects of SPD and multi-scale hybrid reinforcement. Micron-sized Cu particles functioned as a skeletal framework to enhance load transfer efficiency, while nano-Ti particles contributed to grain refinement through Zener pinning and Orowan strengthening mechanisms, which effectively impedes dislocation motion. Coarse Al powder domains formed soft CG zones to accommodate strain, collectively establishing a heterogeneous architecture with alternating hard/soft phases.
3. The Al10-5Cu-10Ti-10Al20 composite demonstrated optimal comprehensive mechanical properties, exhibiting UST (267 MPa), hardness (98 HV), and elongation (16.7%) that were enhanced by 193.4%, 226.7%, and 9.9%, respectively, compared to the matrix material. The heterogeneous microstructure simultaneously improved both strength and ductility through stress partitioning and dislocation blocking mechanisms.
4. Future research should focus on optimizing processing parameters along with post-treatment strategies to achieve balanced reinforcement distribution and interfacial bonding strength, thereby facilitating industrial implementation. This study proposes a novel design strategy for high-performance AMCs, demonstrating the feasibility of synergistic optimization through multi-scale reinforcement and heterogeneous structure engineering.

## Figures and Tables

**Table 1:** Chemical composition of Al 1060 (wt%).

Materials	Mg	Cu	V	Zn	Mn	Si	Fe	Ti	Al
Al 1060	0.03	0.05	0.05	0.05	0.03	0.25	0.35	0.03	Bal.

**Table 2:** AMCs with different reinforcement compositions.

Sample Composition	Fine Al (wt%)	Coarse Al (wt%)	Micro Cu (wt%)	Nano Ti (wt%)	Sample Abbreviation
100 wt%Al	100	0	0	0	100%Al_10_
Al-5 wt%Cu	95	0	5	0	Al_10_-5Cu
Al-5 wt%Ti	95	0	0	5	Al_10_-5Ti
Al-10 wt%Ti	90	0	0	10	Al_10_-10Ti
Al-15 wt%Ti	85	0	0	15	Al_10_-15Ti
Al-5 wt%Cu-5 wt%Ti	90	0	5	5	Al_10_-5Cu-5Ti
Al-5 wt%Cu-10 wt%Ti	85	0	5	10	Al_10_-5Cu-10Ti
Al-5 wt%Cu-10 wt%Ti-10 wt%Al	75	10	5	10	Al_10_-5Cu-10Ti-10Al_20_
Al-5 wt%Cu-10 wt%Ti-15 wt%Al	70	15	5	10	Al_10_-5Cu-10Ti-15Al_20_

**Table 3:** Density and relative density of all samples.

Material	Fundamental Density, g cm^−3^	Measured Density, g cm^−3^	Relative Density, %
Al1060	2.7	2.66	98.5
100%Al_10_	2.7	2.68	99.3
Al_10_-5Cu	2.80	2.68	95.7
Al_10_-5Ti	2.75	2.65	96.4
Al_10_-10Ti	2.81	2.74	97.5
Al_10_-15Ti	2.87	2.73	95.1
Al_10_-5Cu-5Ti	2.86	2.79	97.5
Al_10_-5Cu-10Ti	2.92	2.85	97.6
Al_10_-5Cu-10Ti-10Al_20_	2.86	2.80	97.9
Al_10_-5Cu-10Ti-15Al_20_	2.92	2.83	96.9

## Data Availability

The original contributions presented in this study are included in the article material. Further inquiries can be directed to the corresponding author.

## Abbreviations

The following abbreviations are used in this manuscript:

AMCs	aluminum matrix composites
BM	ball milling
FSP	friction stir processing
CG	coarse-grained
FG	fine-grained
UST	ultimate tensile strength
SPD	severe plastic deformation
SZ	stir zone
DRX	dynamic recrystallization
OM	optical microscope
SEM	scanning electron microscope
EDS	energy-dispersive X-ray spectroscopy
XRD	X-ray diffraction
